# Rapidly Learned Identification of Epileptic Seizures from Sonified EEG

**DOI:** 10.3389/fnhum.2014.00820

**Published:** 2014-10-13

**Authors:** Psyche Loui, Matan Koplin-Green, Mark Frick, Michael Massone

**Affiliations:** ^1^Program in Neuroscience and Behavior, Music, Imaging, and Neural Dynamics Laboratory, Department of Psychology, Wesleyan University, Middletown, CT, USA

**Keywords:** epilepsy, music, seizure, signal detection theory, learning, psychophysics, signal processing, sound design

## Abstract

Sonification refers to a process by which data are converted into sound, providing an auditory alternative to visual display. Currently, the prevalent method for diagnosing seizures in epilepsy is by visually reading a patient’s electroencephalogram (EEG). However, sonification of the EEG data provides certain advantages due to the nature of human auditory perception. We hypothesized that human listeners will be able to identify seizures from EEGs using the auditory modality alone, and that accuracy of seizure identification will increase after a short training session. Here, we describe an algorithm that we have used to sonify EEGs of both seizure and non-seizure activity, followed by a training study in which subjects listened to short clips of sonified EEGs and determined whether each clip was of seizure or normal activity, both before and after a short training session. Results show that before training subjects performed at chance level in differentiating seizures from non-seizures, but there was a significant improvement of accuracy after the training session. After training, subjects successfully distinguished seizures from non-seizures using the auditory modality alone. Further analyses using signal detection theory demonstrated improvement in sensitivity and reduction in response bias as a result of training. This study demonstrates the potential of sonified EEGs to be used for the detection of seizures. Future studies will attempt to increase accuracy using novel training and sonification modifications, with the goals of managing, predicting, and ultimately controlling seizures using sonification as a possible biofeedback-based intervention for epilepsy.

## Introduction

Since, Hans Berger recorded the first human brainwaves in 1924, electroencephalography (EEG) has established itself as one of the most useful non-invasive methods for clinical and scientific investigations of the brain. EEG offers high temporal resolution in investigating how electrical activity of the brain relates to cognition, sleep, emotion, and various neuropathologies such as dementia and epilepsy. Data from an electroencephalogram are typically represented visually, with time and voltage fluctuations on the *x*- and *y*-axes, respectively. In this study, we seek first to represent EEGs from normal and pathological brain rhythms in the auditory modality. Having defined a simple sonification algorithm for EEGs, we show that naïve human listeners can learn to distinguish epileptic seizures from normal brain rhythms using audition alone.

Sonification, in the case of EEG, refers to a process of data-driven sound composition that aims to make certain characteristics of the EEG waveform perceptible (Kramer, 1994). Techniques are being developed for both on-line and offline applications, in the scientific and artistic disciplines (Väljamäe et al., 2013). Sonification is being tested for a wide range of uses including monitoring of biological signals (Glen, 2010), diagnostic work (particularly in cases of epileptic seizure) (Khamis et al., 2012), auditory feedback of motion (Cheng et al., 2013), neurofeedback (McCreadie et al., 2012), and musical composition (Arslan et al., 2005). Here, we present a simple EEG-to-sound mapping algorithm and investigate its potential in monitoring EEGs by determining whether a non-expert population can use these sonifications to detect a seizure, based purely on basic musical comprehension skills. These results will inform the design of seizure monitoring algorithms that rely on abnormal electrical activity in the brain.

### Advantages of EEG sonification

One might ask what the utility of sonified EEG might be, when compared to the existing standard of visual EEG assessments. Sonification may have unique advantages for monitoring physiological rhythms due to the nature of human auditory perception. Compared to visual perception of EEG data, auditory perception – specifically music perception – may be more suitable for biofeedback therapeutic approaches for three reasons. Firstly, musical sounds and seizure EEGs both have strong frequency patterns; this correspondence offers a natural mapping system in translating EEGs to music. For example, pitch control, volume, and duration of a tone can be determined by any combination of parameters from the EEG data (Kramer, 1994).

Secondly, our ears are constantly open, unlike our eyes, and thus the ear acts as a more natural constant monitor that does not require foveation to function. In conjunction with this ability, human beings are surprisingly adept at focusing on important aural information even in noisy environment (e.g., the cocktail party effect, Arons, 1992). Studies have demonstrated that subjects can perform faster and more accurately at complex monitoring of physiological data when the data were presented sonically rather than by visual display (Fitch and Kramer, 1994; Barrass and Kramer, 1999; Watson and Sanderson, 2004). The authors suggest that this advantage of the auditory system can be explained by the fact that auditory recognition of objects occurs simultaneously in multiple parallel streams, in contrast to the visual system, which processes multiple objects serially. Sonification of EEG would prioritize temporal cues and thus allow persons to detect changes in parallel streams of activity as they occur.

Thirdly, people may find listening to music (especially as generated by their own neural activity) more motivating than visually monitoring their EEGs. The enjoyment of listening to esthetically pleasing sonifications might be an important factor in developing therapeutic uses and in improving the relationship patients have with EEG technology. A pleasant esthetic experience for potential end users of sonification technology is important for clinical utility and should thus be central to our goal. Taken together, while sonification does not add new information *per se*, the advantages to sonifying EEG lie in its user interface and increased usability: e.g., sonifying EEG may direct the user’s attention to features of the EEG that are not as readily available to the eye, and thus future users, who may be epilepsy patients themselves or their caregivers rather than trained experts in reading EEG, might be able to detect seizures with minimal training. Sonification may also increase the options available for future biofeedback interventions.

### Early uses of EEG sonification in sound design

Perhaps due to these natural characteristics of the auditory system as a data monitor, electroencephalography has also been revolutionary in the field of experimental music and sound design. Alvin Lucier’s piece “Music for Solo Performer” (1965) is the first well-documented instance of using an EEG for sonification purposes. His composition used two electrodes, attached to Lucier’s temples, to transmit electrical activity (most notably alpha waves) to microphones placed inside various percussive instruments. The amplified frequencies recorded from the electrodes then caused the instruments to resonate at those same frequencies. This transformation from the electrical waveform of the brain to the acoustic waves produced by a drum’s membrane occurred in real-time and represents one of the earliest successful sonification techniques. By modifying his own state of alertness, Lucier was able to modulate the level of energy in the alpha band, thus changing the levels of sound output. Thus, Lucier used his own music composition as an early biofeedback system.

Since Lucier’s “Music for a Solo Performer,” many composers have broadened the scope and output of similar explorations, looking into making more controlled and tonal sonifications of brain waves as recorded by EEG. Pulling from work done by Dr. R. Furth and E. A. Bevers in the 1940s, Bakerich and Scully filed a patent in 1971 for the “electroencephalophone,” which they originally described as a device that can “enable the user to listen to his own brain-wave generation” (1971). In the same decade, Pauline Oliveros and David Rosenboom became seminal figures in experimental music composition by using the electroencephalophone, and other EEG-based forms of synthesis, in their compositions for sonification purposes, yielding works such as Rosenboom’s “Brainwave Music” (1976) and “On Being Invisible” (1977). Our hope is to draw on this history of ingenuity in experimental music to craft an elegant new system for the conversion of the brain’s electrical potentials into sound, for the purpose of creating positive clinical and esthetic outcomes.

### Approaches in EEG-to-sound parameter mapping

The processes of sonification depend crucially not only on the type of data input but also on the data-to-sound mapping process. If the pertinent data can easily be rendered as a simple variable/time graph such as the typical EEG time-voltage readings, then the signal should be easily translatable into sound. If, however, the data lend itself to simple audification or sonification that adheres to the general acoustic wave formula by displaying some form of periodicity in its sequence, then one must consider how much of the recorded data are significant and how much can be considered noise. In these cases, an algorithm that includes filtering of irrelevant noise and/or specific periodicities is necessary for optimal sonification (Hermann and Hunt, 2011).

The ability of the human auditory system to distinguish multiple voices and instruments from background noise make it well adapted to processing sonified EEG. Methods to sonify EEG data remain relatively unique as some have devised means but no method has shown extreme utility compared to any other. Over the past 10 years, new techniques such as parametric orchestral sonification have arisen that allow for the use of multiple channels of data to be sonified from EEG (Hinterberger and Baier, 2005). These approaches to processing multiparametric data allow for experimentation with parameter mapping, where the researcher can match different parameters of the EEG waveform with auditory parameters, such as pitch, duration, and volume. This level of control surpasses basic audification and allows for composition of novel musical scores from EEG data. Hinterberger and Baier (2005) have demonstrated that this technology can be used in real time, using a sample of 0–40 Hz divided into six frequency bands (namely alpha, beta, gamma, theta, and delta) assigned to individual voices in a musical instrument digital interface (MIDI) device. Subjects were able to control these voices and produce music in real time through a brain–computer interface. Musical compositions, created using similar techniques, have the potential to support clinical applications. In the case of applications toward epilepsy, these parameter mapping techniques may form the basis of monitoring systems that inform caregivers of partial seizures that might otherwise go undetected.

In fact, research is already beginning to show that sonifications are successful at representing important EEG data for diagnostic purposes. Khamis et al. (2012) showed that with limited training, non-experts were able to recognize temporal lobe seizures using audified EEG at a rate comparable to expert technicians using visual displays of the EEG waveform. Khamis’s sonification process required compression of EEG signals over time, and the frequencies were limited to 1–10 Hz range prior to time-compression. Using this algorithm, the authors successfully demonstrated the relative ease of detecting seizures from audified EEG by non-experts with minimal training. However, due to the time-compression inherent in their sonification algorithm, this technique poses challenges for real-time sonification.

In contrast to the time-compressed sonifications of Khamis et al., experiments by Baier et al. (2007) have demonstrated the potential of sonified EEG for diagnosis of epileptic seizure in *real-time* using a process of sonification that does not require compression. Their technique of event-based sonification works on the principle of suppressing background noise and highlighting both normal and pathological rhythms. Identifying epileptic rhythms was accomplished by exploiting amplitude of the waveform and inter-maxima intervals to trigger specific sonic elements such as volume, tone duration, and the number of harmonics. This technique relies on stereotyped EEG rhythms and may not to be used from patient to patient without adjustment. Despite this drawback, this work demonstrates that on-line use of EEG sonification is possible and can exploit the diagnostic advantages demonstrated by Khamis et al. (2012).

While certain EEG parameters are used for sonification by almost all researchers in the field, some groups have developed complex parameter analysis that happens before the data are fed into the sonification algorithm. One universal parameter is the time-frequency dimension, and different projects have found different ways to manipulate this parameter. For example, some research has utilized a sliding-window technique, where data are analyzed in increments of several milliseconds or seconds (Arslan et al., 2005). Although this technique helps remove unwanted artifacts, it introduces latency into the system, thus reducing effectiveness for real-time sonification. Another dimension used in sonification is signal amplitude. As the amplitude of EEG signals corresponds to the firing rates of neurons, this parameter is vital in sonifying ictal brain activity, which manifests itself in increased firing rate (Blumenfeld, 2003). Because of the high level of background noise during normal brain activity, measures must be taken to attenuate this noise if a system attempts to diagnose an epileptic seizure. Noise may include intense spiking caused by jaw clenching or head moving, along with other artifacts. For example, some groups have found success by linking extreme maxima and maxima values to separate noise from target activity (Väljamäe et al., 2013). Aside from the time-frequency dimension and amplitude dimension, various groups have used high-level processing of EEG data pre-sonification. These processes include, but are not limited to, quadratic distance in the feature space (McCreadie et al., 2012), Gaussian kernel based on a normal distribution (Hermann et al., 2008), and calculation of time-domain parameters (Hjorth parameters, Miranda and Brouse, 2005).

In summary, current EEG sonification applications can be placed on continuum from *functional* to *esthetic* (Väljamäe et al., 2013). While our work certainly lies on the former side, we would like to make our sonifications esthetically pleasing as well, for two main reasons. First, if an EEG sonification system is to be implemented in a public setting (e.g., a hospital or nursing home), sonifications that are dissonant and cacophonous could be undesirable. Second, creating sonifications that sound pleasing may help non-experts hear fluctuations that correspond to seizures; the more strange and unfamiliar our sonifications are, the harder it will be for someone to hear important developments in the sonified score. Thus, while the clinical outcome of seizure detection is undoubtedly the central goal of the present research, the creation of esthetically pleasing sonifications will serve the clinical goal, as sonifications will be far easier to use as a clinical device over extended periods of time if they sound pleasing, i.e., if they adhere to perceptual and cognitive principles that underpin our appreciation of music [see Lerdahl (1992)]. Thus, our aim is to develop, and test, an algorithm for real-time EEG sonification that provides an esthetically pleasing perceptual experience, while being functionally diagnostic of seizures by a listener with minimal training.

### Goals of the present study

In the present study, we asked whether human listeners with no specialized knowledge of epilepsy, and no previous training in seizure detection, could identify seizures using the auditory modality alone. We presented sonified EEG recordings to a group of naïve listeners in a pre-post-training paradigm. We aimed to test average, non-trained subjects rather than trained experts (1) to eliminate the variable of how much experience the subject has had with using EEG or with seizures and epilepsy, and (2) to see if these non-experts could, given minimal instruction, learn to detect seizures quickly, possibly quicker than if they were learning to detect seizures visually. First, the subjects listened to EEG sonifications of both normal EEG patterns and patterns that correspond to ictal activity. After each trial, the subjects must decide whether the sonification that they just heard corresponds to normal or ictal activity in a two-alternative forced choice test. Then, subjects will receive a short training session on recognizing the auditory patterns that correspond to seizure activity, after which the subjects will take a test similar to the one administered before training. We expect that the ability to differentiate between ictal and baseline patterns of activity will be strengthened by the training session. Performing the pre-training test will help assess the efficacy of the training session, and provide the opportunity to determine what other factors (e.g., musical background, pitch-discrimination ability) might affect the ability to discriminate changes in EEG sonifications.

Our experiment differs from Khamis et al. (2012) in two important ways: (1) we use a sonification algorithm that does not require compression of EEG data, and (2) we use a very short training procedure to determine the shortest possible amount of training needed to perform above-chance levels of seizure detection. By not being restricted to compressed data, our sonification algorithm can be implemented for real-time analysis. Considering that seizure diagnosis is time-sensitive, the ability to sonify EEG data in real-time is not only desirable but also vital to the original goal of sonification.

To summarize, we predict that by listening to sonified EEGs generated by our sonification algorithm, human listeners will be able to identify seizures from baseline, non-seizure activity using the auditory modality alone, and that accuracy of seizure identification from will increase after a short training session.

## Materials and Methods

### EEG database

The electroencephalography data used for this study were accessed through the Children’s Hospital Boston-Massachusetts Institute of Technology (CHB-MIT) Scalp EEG Database (Shoeb, 2009). The EEGs were recorded from pediatric epilepsy patients with intractable seizures. Recordings in the database came from 22 subjects, 5 males, ages 3–22; and 17 females, ages 1.5–19. All EEG data were recorded at 256 Hz, with 16-bit resolution, and spanned values from −800 to 800 mV. Data from both ictal and normal activity were downloaded as European data format (EDF) files, and 10 s sections of data were converted into individual text files in MATLAB.

### Conversion

EEG data were downloaded in EDF from the CHB-MIT Scalp EEG database, which contained information concerning the recordings of the patient including whether the recording contained a seizure, when during the recording the seizure began and ended (in seconds), a listing of all the EEG channels, and the sampling rate (256 Hz) and the total length of the recording. We used 58 files containing seizure and baseline episodes of EEG recordings from 16 patients. For each patient, four EEG recordings were used, two non-seizure and two seizure. For each EEG recording, the Fz-Cz channel was selected as it was closest to Cz, which was listed as the most common electrode position used for sonification purposes (Hinterberger and Baier, 2005). Within the Fz-Cz channel recording, we isolated a 10-s epoch of EEG data that contained a seizure of at least 20 s (for the files that contained seizures), and a temporally matched 10 s epoch of EEG data that contained no seizure (for the files that contained no seizures), while avoiding pre-ictal epochs in the seizure EEGs, and epochs in the non-seizure EEGs that contained obvious EEG artifacts such as those resulting from movement. This resulted in 10 s clips of sonifications corresponding to 2560 points of data at the sampling rate of 256 Hz. EDF files were read using Matlab r2014a and the script edfread (http://www.mathworks.com/matlabcentral/fileexchange/31900-edfread). A Matlab script was then written to read the EDF files, select a 2560 value sequence and write those values, in order, in a new text file. The format of the text file was determined so that the COLL object in Max/MSP 6.1 could sequence the values. Each seizure sonification was created from a text file based off of data 10 s (2560 samples) into the seizure such that the initial stages of the seizure were not used for sonification purposes.

### Sonification

**Figure 1:**
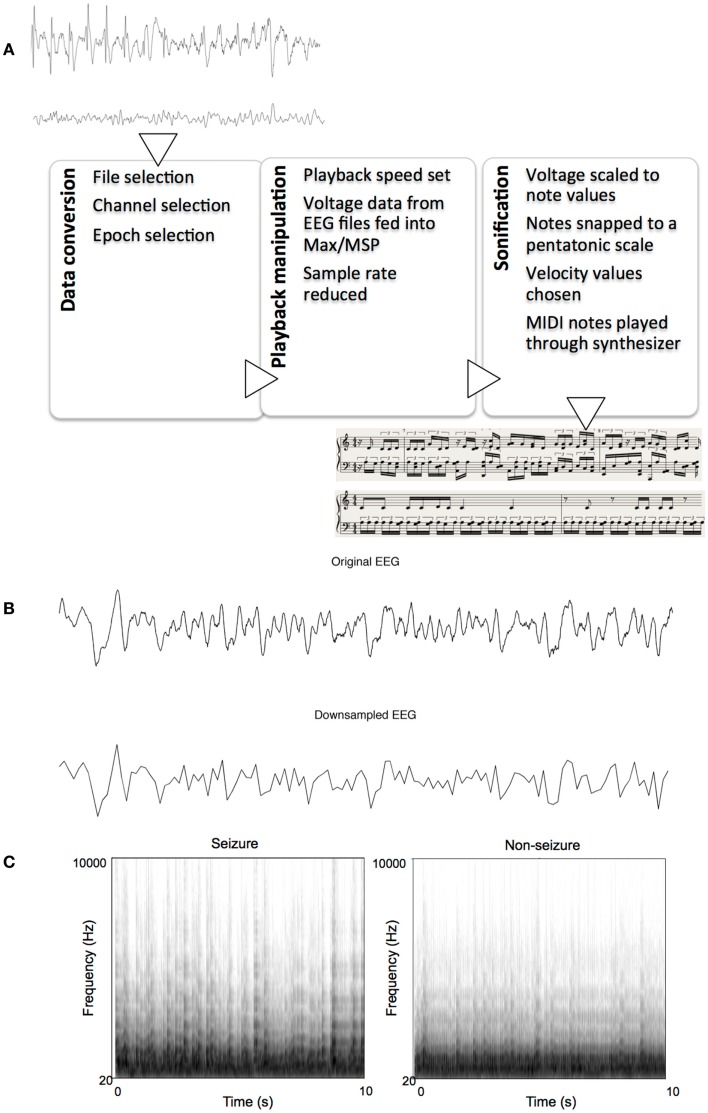
**The sonification process**. **(A)** Flowchart of sonification process, showing seizure and non-seizure EEGs, and their respective sonified scores, **(B)** Example of a 10-s seizure EEG epoch before and after down-sampling, **(C)** Example spectrograms of sonified EEGs: one seizure and one non-seizure.

Each text file from MATLAB contained an array of 2560 indexed millivolt value that corresponded to that 10 s segment. These text files were then imported into Max/MSP for sonification. We constructed an algorithm within Max/MSP for assigning note values to the imported data points using various objects already available within the Max software. The sonification algorithm read every 20th data point in each set, effectively reducing the sample rate to 12.8 Hz, with the results resembling that of a low-pass filter. Examples of the pre- and post-downsampled data are shown in Figure 1B. The numerical data were then scaled linearly to values between 1 and 40 using the “scale” object in Max. These data were then fit to the nearest respective integer scale degree value that corresponded to a major pentatonic scale in the key of C. To do this, the scale degrees corresponding to the C major pentatonic scale up to degree = 40 (i.e., 0, 2, 4, 7, 9, 12, 14, 16, …, 40) were mapped out in a separate list, and the scaled data points were then compared to this list of values. A value of, say, 2, would remain a value of 2 because it matches the scale degree, but a value of 11 would be rounded up and outputted as 12 to match a value in the list of scale degrees. These degree values, now fit to (or “snapped” to) a scale, were then sent as MIDI data, to Logic Pro 9.1.8. Velocity values of MIDI notes were then randomized between 85 and 127 for amplitude variation. The midi notes were then played by Native Instruments Massive^®^ wavetable software synthesizer, using a preset patch named “Old and Far Away.” This patch was a combination of three low-pass-filtered sine- and saw-wave oscillators triggered with fast attack and release times. Ten-second sonified segments were saved as 24 bit, 44.1 kHz audio interchange file format (AIFF) lossless audio files. Figure 1 illustrates the sonification pipeline. Examples of seizure and non-seizure audio files are provided on mindlab.research.wesleyan.edu (Figure 1).

### Experimental design

In order to assess the ability of subjects to detect seizures both before and after training, this experiment was composed of three separate blocks. During the first block, subjects would listen to 13 seizure and 13 non-seizure sonifications, and, for each audio file, report whether they thought the sonification corresponded to a seizure or non-seizure. Next, the subjects would undergo a brief training session, during which subjects would listen to six pre-designated training files, three corresponding to seizure activity, and three corresponding to baseline activity. While listening to the randomly presented training files, subjects would be informed as to the identity of the audio files (i.e., seizure or non-seizure), such that the subjects learn to differentiate seizure sonifications from non-seizure sonifications based on audible characteristics. After this training period, subjects would undergo a testing block identical to the first testing block, albeit with a novel set of 26 recordings (13 seizure and 13 non-seizure). This pre-post design allows for the comparison of detection success both before and after training. The order of audio files within each block was randomized for each new participant. In-house code written in Max/MSP was used to conduct the experiment and record behavioral data.

### Subjects

Fifty-two participants from an Introductory Psychology class at Wesleyan University participated in return for course credit. Approval for the participation of human subjects in this experiment was granted by the Psychology Ethics Board of Wesleyan University. Of these 52 subjects, 43 subjects (mean age 19.02, SD: 1.472; 25 females) provided usable data that were included in our analysis. Partial and total loss of data files, due to incorrect saving procedures, resulted in exclusion of eight participants not included in the final analysis. The nineth excluded participant did not complete the task because a personal history of seizures rendered the subject ineligible. Subjects provided basic demographic information via a survey administered prior to testing. The survey solicited data regarding past musical experience and training, as well as history of mental illness and/or cognitive impairment, and language skills. All subjects reported having normal hearing. Participants completed a pitch-discrimination test, the Montreal Battery for Evaluation of Amusia (MBEA), the Harvard Beat Assessment Test (HBAT), the Shipley Institute Living Scale for non-verbal IQ, and the Interpersonal Reactivity Index survey. These data were kept for analysis of possible correlations between specific attribute/abilities and performance on the task.

### Stimuli

For the experimental interface, we used an iMac computer with Sennheiser HD280 Pro headphones and Max/MSP software. Fifty-eight audio clips were generated from sonified EEGs. These included 13 seizures and 13 baseline rhythms for pre-training testing, another 3 seizures and 3 baseline rhythms for training, and another 13 seizures and 13 baseline rhythms for the post-training test. The audio clips were created in Logic Pro 9.1.8, as detailed in the Section “sonification” above. Subjects were allowed to set the volume to a level, they considered comfortable.

### Procedure

The experiment comprised of three phases: pre-test, training, and post-test.

#### Pre-test

For the first testing block, participants were required to listen to the entire 10 s audio clip before entering either an S keystroke, to indicate seizure, or a K keystroke, to indicate non-seizure. After entering each response, participants would then press the spacebar to proceed to the next trial. After 26 trials of the first testing block were completed, the pre-test phase concluded and the participant moved on to the training phase.

#### Training

The training consisted of six trials, three of which were seizures and three were non-seizures. The training interface was designed to be consistent with the appearance of the testing blocks; however, during the training, the participant was informed visually via text on the screen whether the currently presented audio was derived from seizure or non-seizure EEG activity (“This is a seizure” or “This is not a seizure”) Participants proceeded through trials after hearing each 10 s audio clip by pressing the space bar as in the previous block. After the six presentations were complete, participants were informed via on-screen prompt that the training was complete, and the participant moved on to the post-test phase.

#### Post-test

The third and last block was identical in design to the first block, consisting of 26 new sound presentations, 13 of which were seizures, and 13 were non-seizures. Task instructions were the same as the pre-test. Once the 26 trials were completed, the data automatically saved to text files within Max/MSP.

The order of test trials was randomized for each participant. Each stimulus was presented only once and participants were not allowed to repeat individual trials or blocks.

### Data analysis

All data were imported from text files to Excel and SPSS for analysis. We used one- and two-sample *t*-tests with the conventional alpha levels of *p* = 0.05 to determine the significance of the accuracy of both testing blocks. Additionally, signal detection theory was used to assess changes in discriminability from pre-training to post-training.

## Results

**Figure 2:**
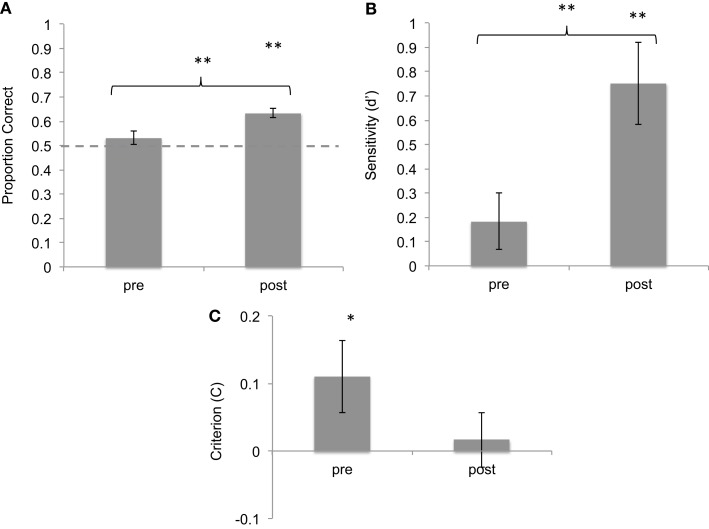
**Results of brief training on seizure identification from sonified EEGs**. **(A)** Proportion correct of seizure identification pre- and post-training, showing improvement after training. **(B)** *d*-Prime values of the same responses, showing improvement in sensitivity. **(C)** Criterion values of the same data, showing reduction in bias. *p < 0.05; **p < 0.01. Error bars reflect between-subject standard error.

Before training, mean accuracy in correctly categorized sonifications was 53.1% (SD = 0.17). This was not significantly higher than chance level [*t*(42) = 1.177, *p* = 0.25, one-sample *t*-test against chance level of 50%]. After training, subjects’ mean accuracy was 63.4% (SD = 0.13). This performance was significantly above chance [*t*(42) = 6.607, *p* < 0.001, one-sample *t*-test against chance level of 50%]. In addition, the difference in average accuracy before and after training was highly significant [*t*(42) = 3.553, *p* < 0.001, two-sample *t*-test; Cohen’s *d* = 0.963] (Figure 2).

Signal detection theory was used to characterize sensitivity and bias before and after training. On average, subjects showed a hit rate of 50% (SD = 24%) and a false-alarm rate of 44% (SD = 18%) before training. After training, the hit rate increased to 63.5% (SD = 17%) and the false-alarm rate was 38% (SD = 18%). The increase in hit rate was statistically significant as shown by a two-tailed *t*-test [*t*(42) = 3.6, *p* < 0.001]. *d*′ measures of sensitivity were calculated for pre- and post-training blocks. Mean *d*′ value pre-training was 0.184 (SD = 0.95) whereas mean *d*′ post-training was 0.751 (SD = 0.75), significantly above-chance level of 0. The difference between these values is statistically significant [*t*(42) = 3,6, *p* < 0.01, Cohen’s *d* = 0.744].

The measure of response bias (C) was used to compare the response criteria adopted by subjects before and after training. Subjects showed a mean positive C of 0.11 (SD = 0.34) before training, which was slightly but significantly above chance [one-sample *t*-test against chance level of 0: *t*(42) = 2.06, *p* = 0.046], confirming a slight response bias toward identifying most sounds as seizures. However, after training, subjects showed a mean C of 0.017 (SD = 0.26), not different from chance level of 0 [*t*(42) = 0.41, n.s.], suggesting a reduction in response bias (Cohen’s *d* = −0.439). Taken together, these data show that with a brief training session, subjects learned to discriminate between seizure and non-seizure sonifications with increased accuracy, greater sensitivity, and reduced response bias.

The data collected in the surveys and preliminary tests were correlated with the recorded accuracy in both blocks as well as the difference between training blocks. No significant correlations were found.

## Discussion

In this study, we defined an algorithm for sonifying seizure and non-seizure EEGs and showed that with a small amount of training, a non-expert population can detect the difference between seizure and non-seizure sonifications with above-chance accuracy. Prior to training, the seizure and non-seizure sonifications were not easily differentiable based on audible features, given that subjects did not perform better than chance on the first block. After a very short training session, however, subjects performed significantly higher than chance, with the mean accuracy rising from 53.1 to 63.4%. The *d*′ values, indicating the sensitivity index, demonstrate that significant improvement in successful discrimination between seizures and non-seizures occurred after brief training. The 10.3% accuracy increase from block 1 to block 2, while statistically significant, is not a high enough level of seizure identification required for the successful future application of sonification technology. However, future experiments will manipulate factors including, but not limited to, length of training, sound design, and training parameters.

Some variables were not controlled for in this experiment. For example, the subject pool was composed of university students of a particular age group, and this pool does not represent the broad range of people that would potentially use this technology. In addition, the amount of subjects’ prior knowledge on epilepsy, EEG, or neuroscience in general was not assessed, although subjects were screened for personal histories of neurological disorder. All subjects were taking an introductory psychology course at the time of participation, and were unlikely to have encountered knowledge on epilepsy from their coursework thus far. Nevertheless, some subjects may have had some familiarity with the neurological correlates of epilepsy, and therefore, possibly better able to detect seizure sonifications from non-seizure sonifications, than others.

In addition, unaccounted-for errors may have occurred during the course of experimentation. For instance, subjects may have pressed the incorrect keystroke due to confusion, which leads to a mismatch between intended and recorded response. Also, due to technical errors, the experiment program did not save input values for 8 of the 52 subjects, and these subjects were not included in statistical analysis.

One identified source of error was the use of an incorrectly labeled audio file in the training patch. When the experiment was completed, it was determined that one of the seizure files, used for training, was sourced from a section of EEG that may not have included a full seizure. This file has been replaced in the latest version of our experiment design. It should be noted that, despite this ambiguity, the training was still successful. We can surmise that had this error been detected sooner, or if it had never occurred at all, the accuracy post-training would only have been increased.

In future studies, we hope to improve and refine the sonification algorithm and its execution. We purposely crafted a simple method to allow development, once we learned more about how subjects responded. Currently, the note is influenced by the waveform and volume is determined by the currently randomized velocity parameter of the MIDI note and could easily be mapped to a relevant parameter of the waveform. There are two separate approaches that can complement one another differently depending on implementation. The first approach is the way the data are translated into a MIDI signal and how much information the MIDI signal contains. Note value, velocity, duration of the note, and other CC values can be mapped into each note base on different parameters. On the synthesis side, we can decide how to route all the MIDI information such that the velocity of a note could correspond to the frequency of a filter, the dry/wet ratio of an effect signal, or volume, in its most simple application. The relative or absolute value of the point in the algorithm could also map velocity, reverb, phasing, and other sound characteristics. Further velocity or other MIDI messages could communicate the relative amplitude or change in amplitude or frequency envelope information. In addition, more waveform analysis could be implemented so that rather than regularly sending notes at the rate of polling (12.8 Hz), notes would be triggered only whenever a peak or trough occurs. Alternatively, a function to eliminate redundant notes series could eliminate uneventful data such that our sonification algorithm consistently causes the intervallic leaping behavior, we have seen. This would mean that extraneous data of normal activity would largely result in very few to no sounds and the sounds would be discrete blips rather than trains of temporally sequenced notes. When seizure activity occurs, the range of values would cause a series of intervals to play. Lastly, future manipulations with the timbre of our instrument may help illustrate the motion of the signal in an obvious way while playing a series of rapid notes with no distinct attack rather than notes, which have a attack such as our current instrument.

Another important area of development is to move toward the goal of a real-time system, i.e., shortening the time delay between the EEG recording and sound playback. We are currently using offline-collected data because of its availability and its validated distinction between seizure and non-seizure categories of EEGs. However, our sonification system is designed to enable a transition toward real-time use as it functions without use of time compression. Furthermore, the sonification algorithm, written in readily available software packages (Max/MSP and Logic Pro), generates sounds in real time as it is reading the EEG data, rendering it flexible toward real-time sonification and platform independence.

Another set of possible modifications to the current experiment involves enhancing or modifying the training period. Whether through visual aids, increased exposure, or repetition of training, the key to successful training will be a balance between maximizing the utility of esthetic interpretation and perfecting a training paradigm for the system. It is our intention that this work will culminate in the creation of pipeline between sonification and EEG recording technologies, allowing for a truly real-time device that may be usable for biofeedback/neurofeedback-based interventions.

## Conclusion

In this experiment, we explored the possibility of sonifying electroencephalogram data for the purpose of seizure detection. By developing a pipeline for data importing and sonification, we constructed an audible representation of cortical electrical activity from seizure and non-seizure EEGs and showed that naïve listeners, independent of musical training, were able to learn to discriminate between baseline and seizure EEG rhythms. Participants performed at chance rates prior to training, but after a brief (1 min) training session, participants improved significantly in both accuracy and sensitivity. These results are concurrent with similar studies and advances in the field of sonified EEG recordings. Further studies will focus on refining the sonification algorithm to optimize auditory analysis for clinical applications. It is our hope that this research will help to create an EEG sonification device that could be useful in a noisy clinical environment, as an alternative to visual EEG monitoring, or in a home environment, to allow for mobile monitoring by non-expert caregivers, as well as future biofeedback/neurofeedback-based interventions.

## Conflict of Interest Statement

The authors declare that the research was conducted in the absence of any commercial or financial relationships that could be construed as a potential conflict of interest.
